# Supplementary material for: Neural correlates of the improvement of cognitive performance resulting from enhanced sense of competence: A magnetoencephalography study
Source: PLoS One. 2021 Jul 23;16(7):e0255272. doi: 10.1371/journal.pone.0255272 (PMC8301610; doi:10.1371/journal.pone.0255272)
Supplement: Attachment — Submitted filename: Response to Reviewers.docx [file pone.0255272.s002.docx]

**Responses to the comments**

**Reviewer 1**

1. MEG data analysis might have be conducted in 15 participants. In this situation, the results of subjective motivation, task performance, and these correlations including the neural activations of right IFG (BA47) and left precuneus (BA7) should be mentioned and discussed.

**Response:**

**The MEG data from four participants were excluded because the numbers of the trials of their MEG data after the removal of the trials contaminated with artifacts were not sufficient for the analysis as described in the Results (Page 13, lines 7 to 11, in the revised manuscript) and therefore, the MEG data analysis have been conducted in 16 participants. Since we were interested in the neural activity related to the increased task performance caused by an academic reward such that induce a sense of competence, we performed the correlation analysis of the neural activity with the alteration of the task performance (i.e., the correction rate in this case) and discussed the supposed neural mechanisms by which task performance is enhanced by academic rewards based on the results of this correlation analysis. As the Reviewer 1 suggested, we added the correlation analysis of the neural activity with the alteration of the subjective motivation; however, we were not able to detect the alteration of the neural activity that correlated with the alteration of the subjective motivation caused by the academic reward. We have discussed the possibility that the neural activity related to the increase in motivation might have been missed in the present study in the Discussion (Page 16, lines 20 to 23, in the revised manuscript). We added the expression, “****i.e., the MEG data were analyzed for 16 participants”, in the Results (Page 13, lines 7 to 8, in the revised manuscript). We added the sentences, “****As an additional analysis, the correlation analysis of the neural activity with the alteration of the subjective motivation was performed: The statistical threshold for this additional analysis was also set at *P* < 0.025”, in the Materials and methods (Page 9, lines 2 to 4, in the revised manuscript) and “The alteration of the neural activity that correlated with the alteration of the subjective motivation were not observed”, in the Results (Page 13, lines 3 to 4, in the revised manuscript).**

2. In Figure 5, it is difficult to identify or visualize the activated regions of this SPM map, and thus, I recommend use other versions of map such as a render map.

**Response:**

**Thank you for the suggestion. We revised the Figure 5 as the Reviewer 1 suggested.**

**Reviewer 2**

1. It was not clear to the reader what the implications of these data were for the academic setting.

**Response:**

**Thank you for the useful comment. We added the sentence, “O****ur finding that cognitive performance can be increased by academic rewards through enhanced activation of task related brain regions would motivate further studies to clarify the neural mechanisms by which academic performance is improved by enhanced motivation to learn in longer time span, leading to establish scientific basis for the educational methods aimed to enhance motivation to learn and to devise effective educational methods”, in the Discussion (Page 17, lines 9 to 14, in the revised manuscript).**

2. The 1-back Stroop task is an interesting activation task. What is the justification for its use to evaluate motivation and academic performance? Has this task been validated previously using behavioral measures related to academic performance specifically?
**Response:**

**We needed a cognitive task with which the improvement of the task performance caused by academic rewards can be evaluated in our present study. We have been using a type of 2-back task as the cognitive task to induce mental fatigue in our past studies investigating the neural mechanisms of fatigue and have realized that it is difficult to evaluate the slight alteration of cognitive performance, such that caused by academic rewards, using by the task performance of the 2-back task. Therefore, we have developed and tried several cognitive tasks by ourselves and finally selected the 1-back Stroop task as the cognitive task which would be able to evaluate the slight alteration of cognitive performance caused by academic rewards. To the best of our knowledge, there have been no reports in which 1-back Stroop task was used to assess behavioral measures related to academic performance. Therefore, one of the aims of our present study was to examine whether the 1-back Stroop task is able to evaluate the alteration of cognitive performance caused by academic rewards. We added a sentence in the Introduction as follows: “The aim of this study was to examine whether the 1-back Stroop task we have developed is able to evaluate the alteration of cognitive performance caused by academic rewards and to clarify the neural correlates of the enhanced cognitive task performance resulting from academic rewards that induce a sense of competence” (Page 4, lines 7 to 10, in the revised manuscript).**

3. Why were only male subjects studied here? This was a study of healthy individuals. What is the justification for exclusion of females in this study? This is a major concern for a study such as this in the 21st century.
**Response:**

**The main reason that the participants of our present study were male was to minimize the magnetic noises caused by metallic components of cosmetics, accessories, closes, and so on. We have conducted several MEG studies with female participants because the purpose of these studies was to examine some kinds of neural activity in female participants and found that even though we have carefully announced to the participants that they should not wear makeup, accessories, and closes containing metallic components, including underwear, the MEG data from more than half of the female participants were contaminated with magnetic noises from unidentified sources, which were thought to be the metallic components included in cosmetics, accessories, closes, and so on: Unfortunately, since our MEG facility have no changing room and the space for removing and re-wearing makeup, we are not able to identify the source of the magnetic noises by removing potential sources of the magnetic noises. In contrast, it is rare for male participants to ware makeup, accessories, and closes containing metallic components, which are the potential sources of magnetic noise. Therefore, we decided to recruit male participants in this present study and planned to perform another study in the future including female participants to generalize our findings if our present experimental design was effective for clarifying the neural mechanisms by which cognitive performance is improved by academic rewards in male participants.**

**We understand the concerns raised by the Reviewer 2 and the editor that it is desirable to include female participants in the human studies in the 21^st^ century. However, we do not have means to perform additional experiments with female participants at this time because the MEG experiments with human participants have been stopped in our university since April in 2020 due to the expansion of COVID-19 infection in Japan, and in addition, our funded project is finished.**

**We would like to point out that although our findings may not be generalized to the population as described in the Discussion (Page 16, lines 18 to 20, in the revised manuscript), we believe that our finding that cognitive performance can be increased by academic rewards through enhanced activation of task related brain regions is fundamental to establish scientific basis for the educational methods aimed to enhance motivation to learn and have great value to motivate further important studies such that clarify whether there are differences between male and female participants in the neural mechanisms of the improvement of cognitive performance caused by enhanced motivation to learn.**

4. Stimuli. ‘…one of four words (Red, Blue, Yellow, or Green in Japanese)…’ What was the stimuli? Were these Kanji pictograms or Kana characters etc? ‘…the color of the present word…’ Do you mean the color in which the word appeared in? What was the visual angle subtended by the stimuli (horizontal & vertical)?

**Response:**

**The words presented in Japanese were Kanji pictograms. We revised the description in the Materials and methods as follows: “Red, Blue, Yellow, or Green in Japanese Kanji pictograms” (Page 5, line 25 to page 6, line 1 and page 30, line 6, in the revised manuscript). We also revised the description, “the color of the present word”, to be “****the font color of the word presented at the time” (Page 6, line 5 and page 30, lines 8 to 9, in the revised manuscript). The visual angle of the Kanji pictograms projected on the screen was 5.7° × 5.7° (horizontal × vertical). We added the description, “The visual angle of the Kanji pictograms projected on the screen was 5.7° × 5.7° (horizontal × vertical)”, in the Materials and methods (Page 6, lines 2 to 3, in the revised manuscript).**

5. Control task. ‘…image indicating the temperature of the fingers was the same for every participant…’ How was the image changed during the course of the task to make the control task seem real for the subject?

**Response:**

**The image indicating the temperature of the fingers was the same during the course of the task. In order to make the participants believe that the temperature of their fingers was actually measured, they were instructed that the bar indicating their temperature corresponded to some range of temperature although the scale and the unit were not shown and therefore, the image indicating the temperature of the fingers might be the same during the course of the experiment even if slight fluctuation of the temperature existed. We revised and added the descriptions in the Materials and methods: “However, in fact, the temperature of the fingers was not assessed during the experiments, and the image indicating the temperature of the fingers was the same for every participant and the same during the course of the task. In order to make the participants believe that the temperature of their fingers was actually measured, they were instructed that the bar indicating their temperature corresponded to some range of temperature although the scale and the unit were not shown and therefore, the image indicating the temperature of the fingers might be the same during the course of the experiment even if slight fluctuation of the temperature existed”, in the Materials and methods (Page 7, lines 6 to 13, in the revised manuscript).**

6. Stimulus presentation software details are missing. What software was used to present stimuli?

**Response:**

**Thank you for the suggestion. We added the sentence, “****Visual stimuli were presented by using OpenSesame software (Version 3.1.7-py2.7-win32; Mathot et al., 2012)”, in the Materials and methods (Page 6, lines 12 to 13, in the revised manuscript).**

**Reference:**

**Mathot S, Schreij D, Theeuwes J. OpenSesame: an open-source, graphical experiment builder for the social sciences. Behavior research methods. 2012;44(2):314-24. Epub 2011/11/16. doi: 10.3758/s13428-011-0168-7. PubMed PMID: 22083660; PubMed Central PMCID: PMC3356517.**

7. Data acquisition details are missing. What was the location of the reference coils? What was the low pass filter setting for data acquisition? High pass is reported, but low pass has been omitted.

**Response:**

**Thank you for the suggestions. The reference sensors were located in the liquid helium dewar, away from participants’ head, in the magnetically shielded room. The original MEG data were high-pass filtered at 0.3 Hz and low-pass filtered at 500 Hz. We added the sentence, “****The reference sensors were located in the liquid helium dewar, away from participants’ head, in the magnetically shielded room” in the Materials and methods (Page 8, lines 8 to 10, in the revised manuscript). We revised the sentence regarding the filter setting for data acquisition to be “The sampling rate was 1,000 Hz and the obtained data were high-pass filtered at 0.3 Hz and low-pass filtered at 500 Hz” (Page 8, lines 1 to 2, in the revised manuscript).**

8. How were eye movements & blinks monitored? These details have been omitted from the manuscript.

**Response:**

**Thank you for the suggestion. We added the description, “****In addition, electro-oculography (EOG) was recorded, from electrodes placed over the left and right ends of the eyebrow of the left eye by using Neurofax 1000 (Nihon-Kohden, Tokyo, Japan) with sampling rate of 1000 Hz applying 0.5 Hz low-cut filter and 120 Hz high-cut filter, over the MEG recordings to help identify the physiological noises caused by eye blinks and eye movements”, in the Materials and methods (Page 8, lines 14 to 19, in the revised manuscript).**

9. How were the fiducials digitized? Polhemus system? These details should be described in the manuscript.

**Response:**

**We did not use specific fiducials-digitize system. As described in the Materials and methods (Page 10, lines 11 to 18, in the revised manuscript), five marker coils were attached to the scalp before the start of the MEG recording and the magnetic field generated by each marker coil was measured during the MEG recording: The position of each marker coil was determined off-line after the recording using a software provided by the supplier of the MEG system (MEG 160; Yokogawa Electric Corporation). During the recording of MR image, five markers, which are detectable in the MR image, were attached to the scalp at just the same points as those at which marker coils were attached during the MEG recording. Then, the MEG data were co-registered onto the MR image based on the locations of these MEG marker coils and MRI markers by using the MEG 160 software. We revised the corresponding description in the Materials and methods to be “The MEG data were superimposed on the MR images using information obtained from these markers and the MEG localization coils, which were attached to the scalp over the MEG recording at just the same points as those at which MR-markers were attached, using the MEG 160 software” (Page 10, lines 15 to 18, in the revised manuscript).**

10. Were ‘empty room’ recordings used to help reject non-brain artifacts? This should be explicitly stated.

**Response:**

**The data obtained by empty room recordings were not used in the present study. We added the description, “The data obtained by empty room recordings were not used”, in the Materials and methods (Page 8, lines 19 to 20, in the revised manuscript).**

11. ‘Epochs of the MEG data, including artifacts, were identified visually and excluded from the analysis.’ What artifacts exactly were excluded from the data? What was the total length of the data epochs? Was the pre-stimulus baseline 300 ms?

**Response:**

**Thank you for the suggestion. We revised the descriptions in the Materials and methods: “Epochs of the MEG data, including artifacts, such that originated from outside the magnetically shielded room remaining after the elimination using reference sensors and physiological noises caused by eye blinks and so on, were identified visually and excluded from the analysis. The total length of the epochs was 250 ms from -300 to -50 ms before the button press as described below” (Page 8, lines 10 to 14, in the revised manuscript).**

12. All version numbers of named software should be included in the analysis.

**Response:**

**Thank you for the suggestion. We added version numbers to all the software described in the manuscript (Page 6, lines 12 to 13; Page 9, line 6; Page 9, line 7).**

13. It is not exactly clear if individual structural MRIs were or were not performed in the subjects. Or was an MRI group template brain was used for the analysis? The description is not clear. If individual subject MRIs were performed in each subject & fiducials were digitized in the MRI images, was the MEG study performed prior to the MRI study to make sure that no additional magnetization was present?

**Response:**

**Individual structural MRIs were performed for each participant. We revised the description regarding MR imaging in the Materials and methods to be “anatomical magnetic resonance imaging (MRI) was performed for each participant using a Philips Achieva 3.0 TX scanner to permit registration of magnetic source locations with their respective anatomical locations” (Page 10, lines 8 to 11, in the revised manuscript). Based on the empirical evidence reported in the literature (Gross et al., 2013) suggesting that the negative effects caused by an MRI scan on MEG data typically disappear within 3 days after the MRI scan, the MEG study was planned to be performed at least 4 days after the MRI study to avoid effects caused by magnetization if the MRI study were not able to be performed before the MEG study. In fact, the interval between the MEG study and the MRI scan was more than 5 days in our present study when the MRI scan preceded the MEG study. We added the description, “****Based on the empirical evidence reported in the literature (Gross et al., 2013) suggesting that the negative effects caused by an MRI scan on MEG data typically disappear within 3 days after the MRI scan, the MEG study was planned to be performed at least 4 days after the MRI study to avoid effects caused by magnetization if the MRI study were not able to be performed before the MEG study. In fact, the interval between the MEG study and the MRI scan was more than 5 days in our present study when the MRI scan preceded the MEG study”, in the Materials and methods (Page 10, lines 18 to 24, in the revised manuscript).**

**Reference:**

**Gross J, Baillet S, Barnes GR, Henson RN, Hillebrand A, Jensen O, et al. Good practice for conducting and reporting MEG research. Neuroimage. 2013;65:349-63. doi: 10.1016/j.neuroimage.2012.10.001. PubMed PMID: WOS:000312283900032.**

14. Statistical analysis overall. It is not clear to me why a generalized overall analysis for main effects and their interactions was not performed in SPM. Main effects of say Task [Stroop, Control] and Iteration [1-4] should have first been performed before resorting to individual t-tests.

**Response:**

**Since we were interested in the neural activity correlated to the increase of the cognitive performance caused by academic rewards, we performed correlation analysis of the MEG data with the improvement of the task performance. Therefore, we performed correlation analyses rather than individual t-tests in the SPM analyses in our present study.**

15. ‘The MEG data were superimposed on the MRI images using information obtained from these markers and the MEG localization coils.’ So what method was used to achieve the co-registration?

**Response:**

**Please refer to the Response to the Reviewer 2’s comment #9.**

16. The reporting of the data should include individual subject data points in the histograms & not just means. Effect sizes should be described together with all results in the text.

**Response:**

**As the Reviewer 2 suggested, we revised Figure 2 and Figure 3 to show individual data points and the effect sizes were added in the Results (Page 12, line 4; Page 12, line 7; Page 12, line 10; Page 12, line 17; Page 12, line 20; Page 12, line 22). We added the description, “Effect size of each paired t-test was calculated by using G*Power software (Version 3. 1. 9. 2; Faul et al., 2007)”, in the Materials and methods (Page 11, lines 8 to 9, in the revised manuscript).**

**Reference:**

**Faul F, Erdfelder E, Lang AG, Buchner A. G*Power 3: a flexible statistical power analysis program for the social, behavioral, and biomedical sciences. Behavior research methods. 2007;39(2):175-91. Epub 2007/08/19. PubMed PMID: 17695343.**

**END**
