# Supplementary material for: Neural correlates of the improvement of cognitive performance resulting from enhanced sense of competence: A magnetoencephalography study
Source: PLoS One. 2021 Jul 23;16(7):e0255272. doi: 10.1371/journal.pone.0255272 (PMC8301610; doi:10.1371/journal.pone.0255272)
Supplement: Attachment — Submitted filename: Responses_Revise_2R.docx [file pone.0255272.s003.docx]

**Responses to the comments**

**Reviewer 3**

1. In page 10, the logical of this sentence, “……caused by magnetization if the MRI study were not able to be performed before the MEG study,” is wrong.

**Response:**

**Thank you for the suggestion. We revised the description to be “if the MEG study were not able to be performed before the MRI study” (Page 10, line 23, in the revised manuscript).**

2. In the result section, I don’t know why authors used the description of “paired t-test without/with the Bonferroni correction”. The Bonferroni correction itself should be performed equally to all the significant test of one study.

**Response:**

**We primarily tested whether the alterations of the level of cognitive performance between task 4 and task 1 were observed in the target or control conditions. In this case, since the paired t-test was performed two times (i.e., for the target condition and for the control condition), the Bonferroni correction was performed. For the additional analysis to confirm the primary analysis, we compared the level of the alteration of correction rate caused between task 4 and task 1 in the target condition with that in the control condition. In this additional analysis, since the paired t-test was performed once, therefore the Bonferroni correction was not performed. Likewise, the analysis which examined whether the alterations of the level of motivation between task 4 and task 1 were observed in the target or control conditions was performed with Bonferroni correction and the analysis in which the alteration of the level of motivation caused between task 4 and task 1 in the target condition was compared with that in the control condition was performed without the Bonferroni correction.**

3. Since, two conditions were performed by all participances. It is difficult to say this kind of experiment design will induce bias. The participance may intend to probe the feedback of “finger temperature and competence of their work” due to the curiosity of why two different experiment conditions they faced with. Which condition was first phase of the experiment of each participants may also affect the outcome of their performance.

**Response:**

**In our present study, the half of the participants performed the target condition first and the other half of the participants performed the control condition first (i.e., the crossover design). While the crossover design has several merits such that being able to avoid problems of comparability of target and control conditions with regard to between-person confounding variables, as the Reviewer 3 suggested, the effects of the academic reward on the cognitive performance might have not been completely the same between the participants performed the target condition first and those performed control condition first; however, since the half of the participants performed the target condition first (group 1) and the other half of the participants performed the control condition first (group 2), our finding that the decrease of the correction rate in the control condition was greater than that in the target condition was not because the target condition was performed first or because the control condition was performed first. In fact, the decrease of the correction rate in the control condition was greater than that in the target condition in both of the groups (the decrease of the correction rate in the control condition over that in the target condition in the group 1 and group 2; mean ± SD: 4.73 ± 5.73 and 1.04 ± 5.80, respectively).**

4. It is also difficult to ensure the improvement of cognitive performance was not the result of the attention which arise by the performance relate feedback. Current control condition is not performance related condition, any performance related feedback may induce similar results like target condition.

**Response:**

**We intended to test whether the improvement of cognitive performance would be caused by the performance related feedback which would work as an academic reward. Therefore, we did not examine whether the improvement of cognitive performance would be caused by the performance related feedback which would not work as academic rewards such that showing images indicating that the performance of each individual in the 1-back Stroop task was equal to or below the average. Since it was shown that the improvement of cognitive performance can be caused by the performance related feedback which would work as an academic reward in our present study, it is of interest to examine whether the improvement of cognitive performance is also caused by showing images indicating that the performance of each individual is equal to or below the average and to clarify the difference in the neural mechanisms that enhance cognitive performance between the condition with performance related feedback which would work as an academic reward and that would not if the cognitive performance is also enhanced by the performance related feedback which would not work as an academic reward in future researches. We added the descriptions, “Since we intended to test whether the improvement of cognitive performance would be caused by the performance related feedback which would work as an academic reward that induce a sense of competence, we did not examine whether the improvement of cognitive performance would be caused by the performance related feedback which would not work as academic rewards such that showing images indicating that the performance of each individual in the 1-back Stroop task was equal to or below the average in our present study. It is of interest to examine whether the improvement of cognitive performance is also caused by showing images indicating that the performance of each individual is equal to or below the average in future researches”, in the Discussion (Page 17, lines 5 to 14, in the revised manuscript).**

5. (Personally, I think the results of cortical source level study based on MEG data is doubtful. I prefer sensor level analysis of MEG.) Authors should also perform sensor level analysis of MEG data to confirm the relation between enhance sense of competence and the improvement of cognitive performance by neural rhythms.

**Response:**

**Since we wanted to clarify precise brain regions correlated with the enhanced cognitive task performance resulting from academic rewards, we planned to perform source reconstruction of the MEG data in our present study. We understand the usefulness of the sensor level analysis of MEG data and that the source reconstruction of the MEG data involves solving ill-posed inverse problem; however, the source level analysis of MEG data is sensitive to the head position in the whole-head MEG helmet relative to the MEG sensors. In our present study, the position of the head in the MEG helmet was not precisely the same between the participants and even within each participant between the conditions because the participants’ heads were outside of the MEG helmet during the rest period between the conditions. Therefore, we think that the MEG analyses with source reconstruction is adequate in our present study. In fact, there are studies reporting that the reliability of the MEG analyses with source reconstruction were comparable or superior to that of the sensor level analyses (for example: Lu et al., 2007; Tan et al., 2015, 2016; Rodriguez-Gonzalez, 2020). We added the description, “We performed source reconstruction of the MEG data because we wanted to clarify precise brain regions correlated with the enhanced cognitive task performance resulting from academic rewards. Since the sensor level analysis of MEG data is sensitive to the head position in the whole-head MEG helmet and the position of the head in the MEG helmet was not precisely the same between the participants and even within each participant between the conditions because the participants’ heads were out of the MEG helmet during the rest period between the conditions in our present study, we think that the MEG analysis with source reconstruction is adequate in our present study”, in the Discussion (Page 17, lines 14 to 22, in the revised manuscript).**

**We also revised the sentence, “A 5-min rest period was instituted between the target and control conditions”, to be, “A 5-min rest period, during which participants’ heads were outside of the MEG helmet, was instituted between the target and control conditions”, in the Materials and methods (Page 7, lines 15 to 17, in the revised manuscript).**

**References:**

**1. Lu BY, Edgar JC, Jones AP, Smith AK, Huang MX, Miller GA, et al. Improved test-retest reliability of 50-ms paired-click auditory gating using magnetoencephalography source modeling. Psychophysiology. 2007;44(1):86-90. Epub 2007/01/24. doi: 10.1111/j.1469-8986.2006.00478.x. PubMed PMID: 17241143.**

**2. Tan HR, Gross J, Uhlhaas PJ. MEG-measured auditory steady-state oscillations show high test-retest reliability: A sensor and source-space analysis. Neuroimage. 2015;122:417-26. Epub 2015/07/29. doi: 10.1016/j.neuroimage.2015.07.055. PubMed PMID: 26216274.**

**3. Tan HM, Gross J, Uhlhaas PJ. MEG sensor and source measures of visually induced gamma-band oscillations are highly reliable. Neuroimage. 2016;137:34-44. Epub 2016/05/08. doi: 10.1016/j.neuroimage.2016.05.006. PubMed PMID: 27153980; PubMed Central PMCID: PMCPMC5405052.**

**4. Rodriguez-Gonzalez V, Gomez C, Shigihara Y, Hoshi H, Revilla-Vallejo M, Hornero R, et al. Consistency of local activation parameters at sensor- and source-level in neural signals. J Neural Eng. 2020;17(5). doi: ARTN 056020**

**10.1088/1741-2552/abb582. PubMed PMID: WOS:000579634100001.**

**END**
