# Supplementary material for: Neural correlates of the improvement of cognitive performance resulting from enhanced sense of competence: A magnetoencephalography study
Source: PLoS One. 2021 Jul 23;16(7):e0255272. doi: 10.1371/journal.pone.0255272 (PMC8301610; doi:10.1371/journal.pone.0255272)
Supplement: Attachment — Submitted filename: Response_PLOSONE_Revise_3.docx [file pone.0255272.s004.docx]

**Response to the comment**

**Reviewer 3**

1. Author answer most of my questions. But on the issue of source level study, as I said, in my opinion the results of cortical source level study based on MEG data is doubtful. Clarify the precise brain region is a good will, however 160 channels of MEG may be can not provide adequate information to reconstruct source level estimation. If authors insist on source level, at least, they should select proper parameters to ensure they could avoid ill-pose issue during the source level studies. Therefore, I suggest authors descript detail of the parameter setting of source level study. And discuss on the aspect of why they set those parameters, only based on other references? Or based on the theory of source reconstruction.

**Response:**

**We used an adaptive spatial filtering method implemented in the software (Brain Rhythmic Analysis for MEG software, BRAM) provided by the supplier of the MEG system (Yokogawa Electric Corporation, Tokyo, Japan) to perform source reconstruction of the MEG data, as described in our previous manuscript. The adaptive spatial filtering method that implemented in the software was a lead-field normalized version of a linearly constrained minimum variance filtering method (Van Veen et al., 1997) and the procedure was optimized for time-frequency source reconstruction (Dalal et al., 2008). The mathematical details of the source reconstruction performed in our present study are present in the previous report (Ueno et al., 2012). The parameter settings of the software were as follows: The active period was “-300 to -50 ms” before the button press in task 4, the baseline period was “-300 to 500 ms” before the button press in task 1, the functional type was {“ERD”, “ERS”}, the functional unit was “F-ratio [dB]”, the voxel spacing was “5 mm”, and the voxel range was “inside the spherical conductor model”.**

**Although, as the Reviewer 3 suggested, the resolution and the accuracy of source reconstructions increases as the number of MEG channels increases (Vrba et al, 2004), it has been theoretically shown that the minimum-variance spatial filter with normalized lead-field has no location bias and this finding was confirmed by the numerical experiments which simulated an MEG system with 148 channels (Sekihara et al., 2005). It has also been demonstrated that the lead-field normalized minimum-variance spatial filter applied to the MEG data recorded by using Yokogawa MEG system (i.e., 160 channels) had high spatial resolution (Sekihara et al., 2005).**

**We revised the descriptions in the Materials and methods to include the details of the source reconstruction: “The adaptive spatial filtering method that implemented in the software was a lead-field normalized version of a linearly constrained minimum variance filtering method (Van Veen et al., 1997) and the procedure was optimized for time-frequency source reconstruction (Dalal et al., 2008). The mathematical details of the source reconstruction performed in our present study are present in the previous report (Ueno et al., 2012). The voxel size was set at 5.0** × **5.0** × **5.0 mm. Under both the target and control conditions, the oscillatory power of the MEG data for task 4 in a 250-ms time window (from –300 to –50 ms before the button press) was calculated relative to that of task 1. The parameter settings of the software were as follows: The active period was “-300 to -50 ms” before the button press in task 4, the baseline period was “-300 to 500 ms” before the button press in task 1, the functional type was {“ERD”, “ERS”}, the functional unit was “F-ratio [dB]”, the voxel spacing was “5 mm”, and the voxel range was “inside the spherical conductor model”” (Page 9, lines 3 to 15, in the revised manuscript).**

**References:**

**1. Van Veen BD, van Drongelen W, Yuchtman M, Suzuki A. Localization of brain electrical activity via linearly constrained minimum variance spatial filtering. IEEE Trans Biomed Eng. 1997;44(9):867-80. Epub 1997/09/01. doi: 10.1109/10.623056. PubMed PMID: 9282479.**

**2. Dalal SS, Guggisberg AG, Edwards E, Sekihara K, Findlay AM, Canolty RT, et al. Five-dimensional neuroimaging: localization of the time-frequency dynamics of cortical activity. Neuroimage. 2008;40(4):1686-700. Epub 2008/03/22. doi: 10.1016/j.neuroimage.2008.01.023. PubMed PMID: 18356081; PubMed Central PMCID: PMC2426929.**

**3. Ueno S, Okumura E, Remijn GB, Yoshimura Y, Kikuchi M, Shitamichi K, et al. Spatiotemporal frequency characteristics of cerebral oscillations during the perception of fundamental frequency contour changes in one-syllable intonation. Neurosci Lett. 2012;515(2):141-6. Epub 2012/04/03. doi: 10.1016/j.neulet.2012.03.031. PubMed PMID: 22465137.**

**4. Vrba J, Robinson SE, McCubbin J. How many channels are needed for MEG? Neurol Clin Neurophysiol. 2004;2004:99. Epub 2005/07/14. PubMed PMID: 16012656.**

**5. Sekihara K, Sahani M, Nagarajan SS. Localization bias and spatial resolution of adaptive and non-adaptive spatial filters for MEG source reconstruction. Neuroimage. 2005;25(4):1056-67. Epub 2005/04/27. doi: 10.1016/j.neuroimage.2004.11.051. PubMed PMID: 15850724; PubMed Central PMCID: PMC4060617.**

**END**
